# High Yield Injection Targets and Danger Zones for Facial Filler Injection

**DOI:** 10.1093/asjof/ojab034

**Published:** 2021-09-22

**Authors:** Christopher C Surek

**Affiliations:** University of Kansas Medical Center, Kansas City, KS, USA

This video will discuss high yield 3-dimensional surface topography landmarks for facial filler injections.^1-13^ Vascular pathways and danger zones will be addressed coupled with key retaining ligament architecture. Injection targets including fat compartments and potential spaces will be outlined in key facial aesthetic subunits, including temple, cheek, lip, and jawline.
